# Supplementary material for: End Sequence Analysis Toolkit (ESAT) expands the extractable information from single-cell RNA-seq data
Source: Genome Res. 2016 Oct;26(10):1397–410. doi: 10.1101/gr.207902.116 (PMC5052061; doi:10.1101/gr.207902.116)
Supplement: Supplemental Material [file supp_gr.207902.116_Supp_Materials.tar.gz › ESAT-esat_v0.1/doc/html/classumms_1_1core_1_1scripture_1_1_build_scripture_coordinate_space.html]

ESAT: umms.core.scripture.BuildScriptureCoordinateSpace Class Reference


ESAT


- Main Page
- Related Pages
- Packages
- Classes
- Files

- Class List
- Class Index
- Class Hierarchy
- Class Members

All Classes Namespaces Files Functions Variables Enumerator Pages

- umms
- core
- scripture
- BuildScriptureCoordinateSpace

Public Member Functions |
Static Public Member Functions |
List of all members

umms.core.scripture.BuildScriptureCoordinateSpace Class Reference

Collaboration diagram for umms.core.scripture.BuildScriptureCoordinateSpace:

[legend]

|  |  |
| --- | --- |
| Public Member Functions | |
|  | BuildScriptureCoordinateSpace (File bamFile, String genomeDir) |
|  | |
|  | BuildScriptureCoordinateSpace (File bamFile, String genomeDir, String outputName, boolean forceStrandedness, TranscriptionRead st, ArgumentMap argMap) |
|  | |
|  | BuildScriptureCoordinateSpace (File bamFile, String genomeDir, String outputName, boolean forceStrandedness, TranscriptionRead strand, String chr, ArgumentMap argMap) |
|  | |
| Map< String, Collection< Gene > > | getPaths () |
|  | |

|  |  |
| --- | --- |
| Static Public Member Functions | |
| static void | write (String save, Map< String, Collection< Gene >> rtrn) throws IOException |
|  | |
| static void | memoryStats () |
|  | |
| static boolean | compatible (Annotation assembly, Annotation read) |
|  | |
| static double[] | l2a (List< Double > list) |
|  | |
| static Map< Gene, Set< Gene > > | getIsoformMap (Collection< Gene > genes) |
|  | |
| static boolean | isoformMapContains (Map< Gene, Set< Gene >> isoformMap, Gene gene) |
|  | |
| static Collection< Gene > | getOverlappingGenes (Gene gene, Collection< Gene > allGenes, double minPctOverlap) |
|  | |
| static Collection< Gene > | getOverlappingGenes (Gene gene, IntervalTree< Gene > allGenesTree, double minPctOverlap) |
|  | |
| static boolean | hasAnOverlappingGene (Gene gene, IntervalTree< Gene > allGenesTree, double minPctOverlap) |
|  | |
| static void | main (String[] args) throws IOException |
|  | |

## Constructor & Destructor Documentation

|  |  |  |  |
| --- | --- | --- | --- |
| umms.core.scripture.BuildScriptureCoordinateSpace.BuildScriptureCoordinateSpace | ( | File | *bamFile*, |
|  |  | String | *genomeDir* |
|  | ) |  |  |

Here is the caller graph for this function:

|  |  |  |  |
| --- | --- | --- | --- |
| umms.core.scripture.BuildScriptureCoordinateSpace.BuildScriptureCoordinateSpace | ( | File | *bamFile*, |
|  |  | String | *genomeDir*, |
|  |  | String | *outputName*, |
|  |  | boolean | *forceStrandedness*, |
|  |  | TranscriptionRead | *st*, |
|  |  | ArgumentMap | *argMap* |
|  | ) |  |  |

Parameters
:   |  |  |
    | --- | --- |
    | bamFile |  |
    | threshold |  |
    | genomeDir |  |
    | outputName |  |
    | forceStrandedness |  |

Here is the call graph for this function:

|  |  |  |  |
| --- | --- | --- | --- |
| umms.core.scripture.BuildScriptureCoordinateSpace.BuildScriptureCoordinateSpace | ( | File | *bamFile*, |
|  |  | String | *genomeDir*, |
|  |  | String | *outputName*, |
|  |  | boolean | *forceStrandedness*, |
|  |  | TranscriptionRead | *strand*, |
|  |  | String | *chr*, |
|  |  | ArgumentMap | *argMap* |
|  | ) |  |  |

Parameters
:   |  |  |
    | --- | --- |
    | bamFile |  |
    | threshold |  |
    | genomeDir |  |
    | outputName |  |
    | forceStrandedness |  |
    | chr |  |

Here is the call graph for this function:

## Member Function Documentation

|  |  |  |  |
| --- | --- | --- | --- |
| static boolean umms.core.scripture.BuildScriptureCoordinateSpace.compatible | ( | Annotation | *assembly*, |
|  |  | Annotation | *read* |
|  | ) |  |  |

static

Parameters
:   |  |  |
    | --- | --- |
    | iter |  |
    | workingAssemblies |  |
    | strand |  |

Returns

Parameters
:   |  |  |
    | --- | --- |
    | iter |  |
    | workingAssemblies |  |
    | strand |  |

Returns
:   Returns true is the exon is a subset of the assembly AND the exon does not match an exon of the assembly

Parameters
:   |  |  |
    | --- | --- |
    | assembly |  |
    | exon |  |

Returns

Here is the call graph for this function:

|  |  |  |  |  |  |
| --- | --- | --- | --- | --- | --- |
| static Map<Gene,Set<Gene> > umms.core.scripture.BuildScriptureCoordinateSpace.getIsoformMap | ( | Collection< Gene > | *genes* | ) |  |

static

Parameters
:   |  |  |
    | --- | --- |
    | genes |  |

Returns

Here is the call graph for this function:

|  |  |  |  |
| --- | --- | --- | --- |
| static Collection<Gene> umms.core.scripture.BuildScriptureCoordinateSpace.getOverlappingGenes | ( | Gene | *gene*, |
|  |  | Collection< Gene > | *allGenes*, |
|  |  | double | *minPctOverlap* |
|  | ) |  |  |

static

Parameters
:   |  |  |
    | --- | --- |
    | gene |  |
    | allGenes |  |
    | minPctOverlap |  |

Returns
:   TODO: optimize using interval tree

Here is the call graph for this function:

Here is the caller graph for this function:

|  |  |  |  |
| --- | --- | --- | --- |
| static Collection<Gene> umms.core.scripture.BuildScriptureCoordinateSpace.getOverlappingGenes | ( | Gene | *gene*, |
|  |  | IntervalTree< Gene > | *allGenesTree*, |
|  |  | double | *minPctOverlap* |
|  | ) |  |  |

static

returns a collection of genes in the interval tree that overlap this gene with atleast minPctOverlap and in the same orientation

Parameters
:   |  |  |
    | --- | --- |
    | gene |  |
    | allGenesTree |  |
    | minPctOverlap |  |

Returns

Here is the call graph for this function:

|  |  |  |  |  |
| --- | --- | --- | --- | --- |
| Map<String, Collection<Gene> > umms.core.scripture.BuildScriptureCoordinateSpace.getPaths | ( |  | ) |  |

Here is the caller graph for this function:

|  |  |  |  |
| --- | --- | --- | --- |
| static boolean umms.core.scripture.BuildScriptureCoordinateSpace.hasAnOverlappingGene | ( | Gene | *gene*, |
|  |  | IntervalTree< Gene > | *allGenesTree*, |
|  |  | double | *minPctOverlap* |
|  | ) |  |  |

static

Returns true if has an overlapping gene with atleast minPctOverlap and in the same orientationin the interval tree

Parameters
:   |  |  |
    | --- | --- |
    | gene |  |
    | allGenesTree |  |
    | minPctOverlap |  |

Returns

Here is the call graph for this function:

|  |  |  |  |
| --- | --- | --- | --- |
| static boolean umms.core.scripture.BuildScriptureCoordinateSpace.isoformMapContains | ( | Map< Gene, Set< Gene >> | *isoformMap*, |
|  |  | Gene | *gene* |
|  | ) |  |  |

static

Here is the caller graph for this function:

|  |  |  |  |  |  |
| --- | --- | --- | --- | --- | --- |
| static double [] umms.core.scripture.BuildScriptureCoordinateSpace.l2a | ( | List< Double > | *list* | ) |  |

static

Converts a list to an array

Parameters
:   |  |  |
    | --- | --- |
    | list |  |

Returns

|  |  |  |  |  |  |
| --- | --- | --- | --- | --- | --- |
| static void umms.core.scripture.BuildScriptureCoordinateSpace.main | ( | String[] | *args* | ) | throws IOException |

static

Here is the call graph for this function:

|  |  |  |  |  |
| --- | --- | --- | --- | --- |
| static void umms.core.scripture.BuildScriptureCoordinateSpace.memoryStats | ( |  | ) |  |

static

|  |  |  |  |
| --- | --- | --- | --- |
| static void umms.core.scripture.BuildScriptureCoordinateSpace.write | ( | String | *save*, |
|  |  | Map< String, Collection< Gene >> | *rtrn* |
|  | ) |  | throws IOException |

static

Here is the caller graph for this function:

---

The documentation for this class was generated from the following file:

- java/umms/core/scripture/BuildScriptureCoordinateSpace.java


---

Generated on Fri Aug 15 2014 15:11:14 for ESAT by  

 1.8.7
